# A Culturally Congruent Psychosocial Intervention for Latino Caregivers of Children with Cancer: Intervention Development

**DOI:** 10.3390/children13030369

**Published:** 2026-03-05

**Authors:** Lessley Torres, Belinda Campos, Haydee Cortes, Sonia Morales, Carol Lin, Lilibeth Torno, Zeev N. Kain, Michelle A. Fortier

**Affiliations:** 1 UCI Center on Stress and Health, School of Medicine, University of California-Irvine, Orange, CA 92868, USA; lessleyt@hs.uci.edu (L.T.); bcampos@uci.edu (B.C.); cortesh@hs.uci.edu (H.C.); zkain@hs.uci.edu (Z.N.K.); 2 Sue & Bill Gross School of Nursing, University of California-Irvine, Irvine, CA 92697, USA; 3 Department of Chicano/Latino Studies, University of California-Irvine, Irvine, CA 92697, USA; 4 Department of Anesthesiology & Perioperative Care, University of California-Irvine, Irvine, CA 92697, USA; 5 Department of Oncology, Rady Children’s Health, San Diego, CA 92123, USA; sonia.morales@choc.org (S.M.); clin@choc.org (C.L.); ltorno@choc.org (L.T.); 6 Department of Pediatrics, School of Medicine, University of California-Irvine, Irvine, CA 92697, USA

**Keywords:** social determinants of health, healthcare disparities, pediatric oncology, community-based participatory research, pediatric palliative care

## Abstract

**Highlights:**

**What are the main findings?**

- This study highlights the need for community-engaged approaches to develop culturally congruent interventions to address disparities in palliative care for marginalized communities.
- Emotional well-being, health literacy, and healthcare that incorporates Latino cultural values emerged as important targets for a behavioral intervention to improve quality of life in Spanish-speaking families impacted by childhood cancer.

**What are the implications of the main findings?**

- Engaging in a community co-development approach results in a culturally congruent intervention that utilizes community and healthcare providers to create a relevant intervention for Spanish-speaking families impacted by childhood cancer.
- Results of this study can serve as a model for development of culturally congruent interventions to address cancer health disparities in children and families.

**Abstract:**

**Background**: Cancer health disparities among Latinos in the United States are pervasive and manifest in higher morbidity and mortality among pediatric cancer patients and their parents/caregivers. There is a need to engage in culturally congruent approaches to develop interventions that effectively mitigate cancer health disparities among Latino caregivers. **Aims**: The purpose of this manuscript is to present the methodological process of adopting a community-based participatory research (CBPR) approach to develop a culturally congruent intervention to address psychosocial cancer health disparities in Spanish-speaking Latino families impacted by childhood cancer. **Materials and Methods**: We established two partnerships with Spanish-speaking parents of children previously treated for cancer and whose children were currently undergoing cancer treatment to collaboratively identify psychosocial intervention targets. A total of 22 meetings were held with community collaborators (*n* = 13) that followed CBPR principles. All meetings were audio recorded, transcribed, and coded using an inductive approach. **Results**: The intervention framework identified three psychosocial outcomes: caregiver health literacy, culturally congruent care, and emotional well-being. A 12-session intervention addressing the three outcomes was developed integrating cultural values and bilingual and bicultural community and healthcare providers. **Conclusions**: A CBPR approach was adopted to address disparities in quality of life in Spanish-speaking caregivers of children with cancer, which resulted in a multicomponent intervention that addresses the informational, practical, and psychosocial needs of Latino caregivers. The intervention can help mitigate disparities in well-being for Latino families impacted by childhood cancer by incorporating culturally relevant strategies to optimize health.

## 1. Introduction

Latinos are the largest minoritized racial/ethnic group in the United States (U.S.), comprising 19.1% the total population [1]. Cancer health disparities among Latinos and other underrepresented racial/ethnic groups are pervasive and include poor health outcomes and higher morbidity and mortality compared to non-Latino Whites [2]. As a note, we recognize that perspectives and preferences vary regarding terms used to reflect the Latino population (e.g., Hispanic, Latino, Latinx, etc.); the Corazones used “Latino” to self-describe and, thus, this term is used throughout this manuscript.

Intersectional approaches are conceptual frameworks first articulated by Kimberlé Crenshaw that recognize the role that multiple social determinants of health, including power and wealth inequalities, play in sub-optimal health outcomes [3]. Consistent with this framework, Latino children face intersecting factors that drive cancer-related health disparities, including economic instability; limited educational attainment, health literacy, and access to care; lack of culturally and linguistically competent healthcare; and systemic racism and discrimination [4]. These disparities extend to parents/caregivers as well, with evidence showing negative impacts on financial status [5], psychological well-being [6,7,8], increased health care use/costs and higher rates of morbidity and mortality [9,10,11,12,13]. Yet, there is limited focus on supportive interventions for caregivers of children with cancer that target the intersecting factors that drive cancer-related health disparities [9], and an extreme dearth of interventions focused on Spanish-speaking Latino caregivers who experience language as an additional barrier in access to care [14].

**Figure 1:**
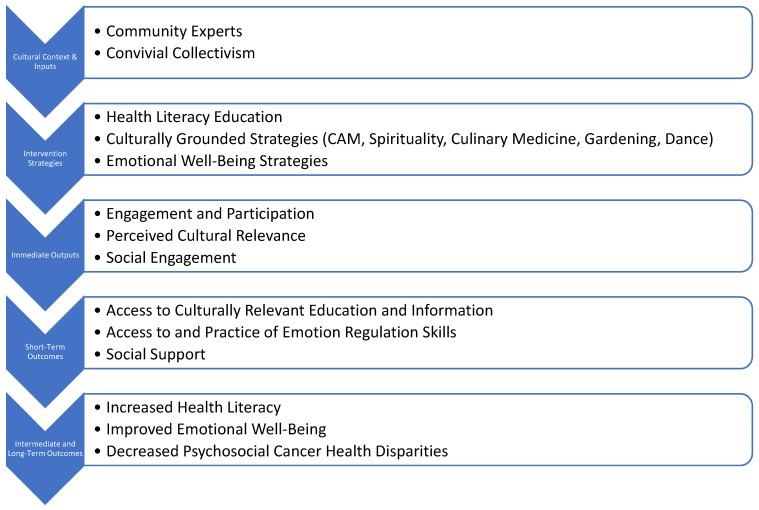
Logic model for intervention approach.

The role of culture in health outcomes is key [15]. A primary cultural context for Latino families is convivial collectivism [16], which involves cultural values that emphasize positive social interactions that avoid negativity (simpatía) [17,18], an emphasis on politeness and avoidance of topics that might cause embarrassment or discomfort (respeto) [19], and prioritization of close family relationships (familismo) [20]. However, Latino families in the US must navigate a biomedical cultural context that is individualistic and prioritizes the self [15]. The theoretical model of convivial collectivism serves as a framework for the development of culturally congruent interventions targeting health outcomes [15]. Culturally congruent care reflects healthcare that is in alignment with cultural values such that it can lead to mitigation of health disparities and improvement in health outcomes. Based upon this framework, we propose a model of how culturally congruent interventions can improve factors associated with health disparities in Latino families with a focus on disparities in emotional well-being and health literacy [21,22]. More specifically, we proposed that incorporating cultural values into a supportive intervention for parents and caregivers will directly address social support and can improve access to health-related information and incorporate culturally relevant emotional regulation skills that will lead to improvements in health literacy and emotional well-being (Figure 1).

**Figure 2:**
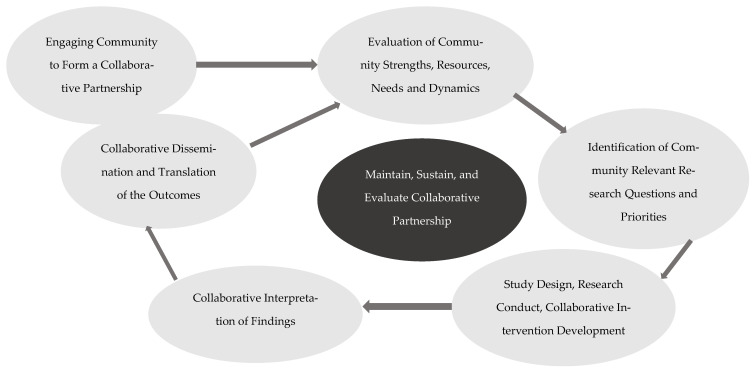
Community-based participatory research components and process.

There are clear methodological paths to address gaps in existing interventions to effectively mitigate health disparities driven by social determinants of health. A recent (2024) review of interventions specifically designed to address racial/ethnic cancer health disparities described 10 studies primarily focused on the Latino population [23]. Notably, none of these interventions addressed Latino caregivers of children with cancer and only five of the 34 manuscripts that met the criteria for the review showed positive impacts of the intervention. Those results highlight the urgent need to develop culturally congruent interventions that effectively address cancer health disparities among Latino caregivers. Community engaged research (CEnR) provides an opportunity to develop culturally congruent interventions by partnering with the community of focus through an iterative process to collaboratively develop an intervention that incorporates feedback from community members to address issues unique to a target population [24]. Community-based participatory research (CBPR) is one such approach that involves developing an equitable partnership between community members and academics throughout a research process [25,26] and has been shown to effectively address disparities, including systemic inequities in access to care and care innovation, and address language and literacy carriers to care [25,26,27]. We assert that adopting a CBPR approach to partner with the Latino community in order to co-develop an intervention is essential to address psychosocial cancer health equity in this population by addressing systemic factors that promote health disparities. CBPR involves an ongoing, collaborative community partnership that involves engaging with community experts in all phases of the research process, from conceptualization of the research questions to development of interventions and dissemination of intervention evaluation (Figure 2). The present manuscript describes the development of the intervention and a subsequent manuscript in this edition describes the formative evaluation and preliminary efficacy of the intervention.

## 2. Materials and Methods

In order to recruit community partners with lived experience in the pediatric oncology setting, we used convenience sampling to recruit families in which parents and children self-identified as Latino, the primary parent(s) language was Spanish, children were diagnosed with cancer, and annual household income was less than $60,000. Eligible parents were identified and recruited from the oncology registry of children currently receiving, or scheduled to receive, treatment at Rady Children’s Health, Orange Campus (CHOC Children’s) Hyundai Cancer Center. Flyers, phone calls, and in-person recruitment were used to recruit members for the community collaboration. This study was approved by the Institutional Review Boards at CHOC Children’s and the University of California, Irvine. All community partners provided informed consent. Consistent with the reporting expectations of the Equator Network, File S1 includes the Consolidated Criteria for Reporting Qualitative Research (COREQ) Checklist [28].

### 2.1. Meeting Design and Procedures (Phases I and II)

The purpose of phase I was to develop the conceptual framework for the intervention with families whose children had completed cancer treatment and the purpose of phase II was to validate the intervention framework for families at the beginning of their child’s treatment (i.e., within 16 weeks of diagnosis). This time window was chosen to ensure that the needs of families who were in the early stages of cancer were adequately represented but that we did not require families who were just diagnosed and likely overwhelmed to participate in an intensive collaborative process.

To remain consistent with the CBPR principles of collaboration, co-learning, and mutual benefit, a series of meetings was held with community partners and academic researchers to develop trust, address questions, and problem-solve challenges regarding optimal psychosocial functioning among Spanish-speaking Latino families impacted by cancer. All academic researchers who were trained in CBPR principles and strategies involved in the meetings (LT and HC, both women and working as project coordinators) were bicultural and bilingual Spanish-speakers. There was no priori relationship between researchers and community partners. Community partners were oriented to the CBPR process and approach at the first meeting and at this meeting, the purposes, goals, and personal interests in the research focus were shared. Thus, all meetings were conducted in Spanish and audio recorded, transcribed, and translated from Spanish to English using a certified translation service and then reviewed by native Spanish speakers to ensure accuracy. Field notes were also taken during the meeting to capture non-verbal expressions and experiences to provide context for the meeting transcripts. To navigate differences between education, class, and immigration status between academic researchers and community partners, we acknowledged power dynamics and prioritized relationship-building with partners. This involved adopting traditional community practices in social gatherings such as “cafecitos”—check ins to facilitate social connections, and “besitos”—a common cultural greeting in Latino communities. Efforts were also made to build trust among community partners through action, to establish shared ownership of the CBPR process (as outlined below with each member assuming an active role), to validate and acknowledge diverse experiences, and to involve community partners in all phases of the research process. Disagreements about the intervention content in community partner meetings and member checks did not arise and all intervention components were developed collaboratively with expressed agreement and validation by both community partners and academic researchers.

Because CBPR emphasizes equitable collaboration, compensating team members for their time and expertise is essential. As Black et al. (2013) note, hourly payment for community partners recognizes their unique contributions [29]. We secured intramural funding through the UCI Institute of Clinical Translational Science CCRI award (UL1 TR001414) to ensure this equity and each individual community partner was compensated $100 for each group meeting. Our collaborative group of community partners and academic researchers created a name for our partnership, which was Corazones Unidos por Una Vida (Hearts United for One Life), henceforth referred to as Corazones.

### 2.2. Academic Team Positionality Statement

The CBPR process was conducted by bicultural, bilingual Spanish-speaking women researchers with experience working in Latino communities. The principal investigator (PI) is a White pediatric psychologist with expertise in community-engaged research addressing health disparities. Although shared language and cultural backgrounds among CBPR and academic team members supported rapport and culturally grounded interpretation, the research team did not share the lived experience of parenting a child with cancer. Additionally, the PI’s racial identity and professional positioning within academic and healthcare institutions reflect forms of structural privilege and institutional authority that shape knowledge production and may influence participant interactions and analytic interpretation. We therefore approached data collection and analysis reflexively, which was guided by a constructivist, community-engaged orientation, while attending to power dynamics, engaging in ongoing critical dialog about positionality, and prioritizing participants’ voices and meanings in representing unmet quality-of-life needs.

### 2.3. Meeting Process (Phase I)

A total of 17 two-hour meetings with community partners were held. The first 9 meetings were held at the hospital where children were receiving treatment. Subsequently, the COVID-19 pandemic forced a shift in the meeting process to synchronous meetings via web-based video conferencing. Although we were unclear initially if this shift would be successful, we found that not only did it maintain the community collaboration, but it also addressed many potential barriers to participation, including transportation and childcare. More specifically, all members reported improved ability to attend meetings virtually and appreciation of the lack of concerns with having to drive or be transported to the on-site meetings and flexibility with childcare needs are a result of virtual attendance. The remaining 8 meetings were conducted via video conferencing.

The initial meetings were exploratory in nature; the academic researchers guided the conversation via open-ended questions to determine areas of importance for community partners. Initial questions asked partners to share their experiences of care during their child’s treatment process and then follow-up prompts inquired about what supportive care was helpful/not helpful or not provided. Participants were asked to share about challenges in access to care with the focus being on family well-being and psychosocial functioning (e.g., What were your experiences during your child’s treatment journey? What would you liked to have known about cancer when your child was first diagnosed? What was helpful in supporting your family during cancer treatment? What challenges did you encounter during your child’s treatment?). Based upon responses from Corazones, the discussions ultimately centered around barriers and challenges to optimal quality of life for families during the treatment process, and protective factors and resources during the treatment process that allowed the collaborative team to identify opportunities for a culturally congruent intervention framework.

### 2.4. Meeting Process (Phase II)

A total of five virtual meetings were held with the second group of community partners that involved presenting the topics, themes, and intervention conceptualization with a goal to gather convergent evidence for the themes that emerged in Phase 1 meetings. Meetings involved presentation of major themes and intervention concepts with open-ended discussion questions to determine whether the intervention concepts were relevant, culturally appropriate, and assess if any intervention targets were missing for families beginning their child’s treatment process. No repeat interviews were required given the data saturation provided by our Phase II group of community partners.

### 2.5. Meeting Roles—Phases I and II

To maintain CBPR’s principles of collaboration and equity, members of Corazones actively rotated meeting roles. Academic researchers served as facilitator, note-taker, and evaluator, while community partners assumed roles such as timekeeper (marca-tiempo), investigator (investigadora), encourager (animadora), and agenda facilitator (facilitadora de agenda). Rotating these responsibilities gave community partners a sense of agency, minimized power differentials between academic and community members, and ensured inclusive discussion by managing time, balancing participation, encouraging quieter voices, and clarifying concepts. This process also established shared norms and infrastructure that supported an equitable, community–academic partnership [25].

### 2.6. Thematic Data Coding (Phases 1 and 2)

Following each meeting, the academic researchers reviewed transcripts of the meeting discussion. Two members of the academic team coded the transcripts for themes using an inductive approach. Textual information was coded using a computer-based system (Atlas/Ti) which allows for easy retrieval of the analytic coding. Data analyses were iterative because qualitative data collection and data analysis occur simultaneously and concurrently. Data were analyzed immediately using constant comparative analysis to systematically code the data. The transcripts of the interviews were reviewed by and discussed with the entire research team, including the community partners. Codebooks were generated, continually updated, and reviewed. Reliability and validity of using this method are also iterative and involved several strategies. We employed ‘member checking’ by presenting results of the coding process to community partners to confirm that the ongoing data analysis/interpretation made sense to them and reflected their perspective. In addition, research team members examined the ongoing data analysis periodically and discussed whether the analysis was supported by the data collected.

## 3. Results

### 3.1. Participants

Participant demographic data for both phases are presented in Table 1.

Phase I: Initial recruitment of community partners involved approaching 16 families, which resulted in a group of primarily mothers (*n* = 13) with a smaller number of fathers (*n* = 2) (93.8% recruitment rate). Of these recruited parents, the two fathers dropped out over time and three mothers dropped out over time due to various reasons including complications of their child’s illness, death of an immediate family member, and time conflicts with the scheduled meetings. The final group comprised eight mothers. The mean age of the community partners was 36.17 (5.91) years. Most (62.50%) reported their country of origin as Mexico, one was born in Honduras (12.50%), one was born in Guatemala (12.50%) and one did not specify country of origin (12.50%). Average annual household income was 20,892.67 (15,600) U.S. dollars, and the average household size was five (1.73) members. Mean number of years of education was 12.75 (3.34), and most (62.50%) members were married or partnered.

Phase II: We recruited an additional five mothers of children who had been diagnosed with cancer in the previous 16 weeks and who were currently undergoing treatment. This group of partners was, on average, 42.20 (2.87) years. The majority (four) immigrated from Mexico, one member was born in Guatemala. Average annual household income was 15,433.60 (8966.61) U.S. dollars, and the average household size was six (1.50) members. Education and marital status were not collected from the phase II partners.

### 3.2. Collaborative Development of the Intervention

No new themes emerged following the 22 group meetings, resulting in thematic saturation of the data. In engaging in the CBPR process with our community partners, three key themes of psychosocial support were identified and included that were consistent with health disparity gaps in the literature [21,22]: (1) a need to address parent and caregiver emotional well-being, (2) lack of health literacy that impacted medical decision-making and navigating a complex cancer healthcare environment, and (3) lack of culturally congruent care (Figure 2). The thematic results of this work and detailed descriptions of each key theme are presented in Table 2 and File S2. Briefly, parents highlighted the lack of access to evidence-based and/or reliable resources to support parent and caregiver emotional functioning (e.g., distress, coping), challenges in making medical decisions and navigating a healthcare environment given lack of resources in Spanish, and healthcare interactions that lacked incorporation of cultural values (e.g., personalismo, confianza, respeto, familismo, simpatía, and tacto—described in detail in File S2) that could lead to mistrust and a lack of informed care. The contrast between cultural values from a convivial context and values in the U.S. healthcare system are presented in Table 3. The results of Phase II validated all the intervention components and strategies and in addition, suggested the need for a focus on spirituality in the intervention. Accordingly, a session with spiritual services was incorporated to reflect culturally congruent care.

**Figure 3:**
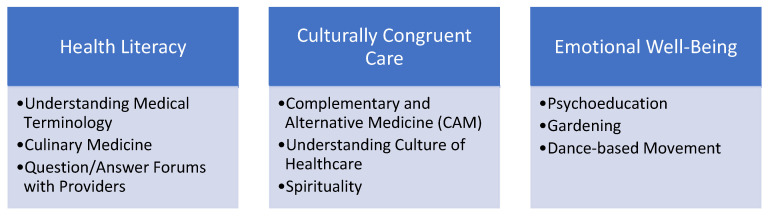
Conceptual model of intervention targets and components.

Each target of the three themes outlined above (Figure 3) is addressed by several relevant strategies that are detailed below. The cultural values described in File S2 were embedded in each of the intervention strategies both by engaging community and hospital partners involved in the intervention creation and delivery who were from the target community (e.g., Latino and Spanish-speaking) as well as developing the intervention in collaboration with the Corazones partners. Specific intervention components are described below and we refer readers to see these components exactly as they were presented to participants in this study in the Supplementary Materials so that readers may evaluate the materials in consideration of their own interventions in similar populations or to assess in consideration of how to tailor materials in their own interventions.

### 3.3. Health Literacy

Understanding medical terminology and navigating the healthcare setting. To address this need, Corazones developed a cancer-specific brochure that distilled the most relevant information for navigating the healthcare system and included areas to note questions for oncology providers, both essential and emergency information, phone numbers to use when care was needed, and information specific to the strategies developed for the Corazones intervention (Supplementary Figure S1). This brochure addresses the expressed needs of the community in ensuring a clear understanding of medical terminology that is commonly used in the oncology setting, treatments and procedures encountered, when and how to contact providers in the case of side effects or adverse events, a place to document specific providers involved in their child’s care, and to note questions to ask of providers and/or at their child’s appointments.

Culinary medicine. To meet health literacy nutritional needs, two components of culinary medicine were developed for the Corazones intervention: a session with dieticians focused on nutrition-related literacy and a cooking class focused on culturally relevant foods that were also appropriate for cancer-related dietary needs. Nutrition-related health literacy included a dietician delivered session on how to read and understand nutrition labels (Supplementary Figure S2) using the Newest Vital Sign [30], which has been used in multiple racial/ethnic groups and has been validated against other measures of literacy including the Test of Functional Health Literacy in Adults (TOFHLA) [30]. For the cooking class, our collaborative team was able to partner with a renowned chef and owner of multiple restaurants in the community to provide a live cooking session using recipes modifying traditional foods (Supplementary Figure S3) that included distributing all ingredients and supplies to families.

Question/Answer forums. To address barriers in access to evidence-based resources and cultural barriers to accessing necessary medical/treatment information in healthcare interactions, we incorporated in person/synchronous question/answer forums with oncology providers, including oncologists, social workers, and case managers. This allowed parents/caregivers to gather necessary information about their children’s diagnosis, treatment, and available resources and have the confidence that the providers are welcoming of questions and providing reliable information in Spanish. Accordingly, we partnered with bilingual and bicultural oncology providers to facilitate these sessions in a group format to provide an opportunity for parents and caregivers to ensure access to relevant, valid, and timely health-related information that also integrated cultural values such as personalismo (an emphasis on warm and trusting personal relationships), respeto (an emphasis on respect, deference, and social hierarchy), familismo (an emphasis on focus on the family unit over individual needs), and tacto (an emphasis on warm and positive social interactions that avoid conflict and/or negativity) to ensure cultural relevancy. Parents/caregivers were provided with an intake sheet that allowed them to introduce questions in advance of the meetings as well as asking questions during the meetings.

### 3.4. Culturally Congruent Care

Complementary and alternative medicine (CAM). To address the need to incorporate a focus on traditional approaches to symptom management and healthcare and in collaboration with the Susan Samueli Integrative Health Institute at UC Irvine, we generated intervention components that equipped parents and caregivers with culturally relevant strategies for pain and symptom management at home. For intervention delivery, we partnered with a bilingual, bicultural Traditional Chinese Medicine trained and certified practitioner who delivered sessions teaching cancer-focused massage, acupressure, and aromatherapy that were able to be taught via video conferencing to parents. These strategies were taught both for self-administration and for parents to administer to their children in order to target parent well-being as well as empower parents with effective strategies to help manage pain and symptoms in their children during cancer treatment. Parents were also provided with CAM reference cards (Supplementary Figure S4) in order to implement strategies outside of sessions.

Understanding the culture of medicine. Due to the lack of *tacto* and *simpatía* observed in interactions with healthcare providers and because of challenges in meaningfully impacting cultural competence using healthcare provider focused interventions, our collaborative group determined this intervention should focus on providing families information and education regarding the culture of medicine to empower navigation of appointments with providers to ensure needs are better met. The rationale behind this approach was so families have an understanding of why patient–provider interactions may not always contain elements valued in Latino culture (*personalismo*, *familismo*, *simpatía*) and to build confidence and efficacy in their ability to manage healthcare provider interactions. This involved developing a contact tree which contains information on the roles of the different providers (e.g., oncologists, nurses, social workers, child life specialists), what parents can expect from each of these providers, and how parents can advocate for their child’s and their family’s needs from various providers (Supplementary Figure S5).

Spirituality. A session with Spiritual Services at CHOC Children’s was incorporated based upon input from the Corazones. For this component, families complete a Spirituality Inventory, which contains questions not only about spiritual beliefs (e.g., “I regularly participate in spiritual activities with people who share my beliefs or have a community with whom I can be open and honest about my beliefs.”), but also about spiritual wellness, including questions such as “I can find peace when I don’t know what is going to happen”, and “I have a sense of belonging, meaning and purpose in life”. This allows for tailoring of the session to family needs and ensuring that relevant topical areas are addressed.

### 3.5. Caregiver Emotional Well-Being

Psychoeducation. To address the identified emotional well-being needs of families, a component of the intervention is sessions delivered by a bicultural, bilingual pediatric psychologist with expertise in psycho-oncology to provide psychoeducation on self-care strategies, emotion regulation for children and siblings impacted by cancer, communication strategies, and when and how to seek psychological resources. Specific topics requested by community members include management of anxiety and treatment-related fear and disclosure of diagnosis to family members and friends. To engage families in emotion regulation strategies, a culturally congruent component that leveraged the popular Loteria game was implemented in which families could share successful use of strategies learned through a culturally relevant game (Supplementary Figure S6). Loteria is a traditional Mexican board game, similar to bingo, and in the context of this intervention was designed for families to check off the various self- and family-care strategies learned and then implemented outside of intervention sessions.

Gardening. To address emotional well-being via an evidenced-based and culturally congruent approach to gardening, consultation with the director of the Master Gardener program at the University of California resulted in the component of a salsa and salad garden, which can be grown year-round and accomplished in a planter box, which is important for families who do not have access to outdoor space (all materials were provided to families). The salsa garden can serve as an evidence-based stress management intervention that also grows healthy, organic vegetables to make traditional Latino foods. The intervention incorporated two gardening sessions that focused on planting and growing (session 1) and harvesting and care/maintaining (session 2) of the salsa and salad garden.

Dance-based movement. To develop a culturally congruent fitness component of dance-based movement, we partnered with Dr. Jan Schroder, Professor of Kinesiology at California State University Long Beach to create asynchronous sessions via a video conferencing platform. This approach to exercise is culturally congruent because it is social and would be accessible and attractive to other family and support members involved in the child’s caregiving (*familismo*) and because the dance-based intervention (i.e., Zumba) could incorporate the preferred music of Latino families. The Corazones chose the music for all the videos, which included dance, stretching, and resistance exercises.

### 3.6. Overall Corazones Intervention

**Figure 4:**
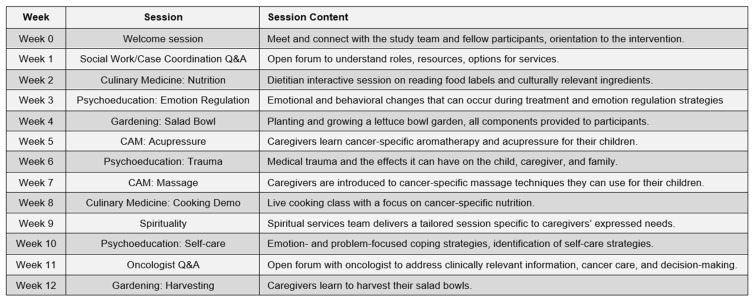
Schedule of Corazones intervention sessions.

Figure 4 presents the totality of the 12 intervention sessions by content addressed in each of the sessions. The intervention was delivered weekly synchronously and then hosted on a website created specifically for the program so that families could securely access all intervention content to review material as needed. The fitness component described above was included as an “on demand” video channel for families to access throughout the intervention. Motivation and encouragement to engage in the videos was supported in the psychoeducation component via the Loteria cards.

## 4. Discussion

This paper reports the process of applying a CBPR approach to understand and mitigate disparities in psychosocial functioning and quality of life in Latino caregivers of children undergoing cancer treatment using a culturally congruent approach. Together, the academic research team and community members developed a collaborative partnership, identified barriers to optimal psychosocial functioning for Spanish-speaking families, and conceptualized components of an intervention that target three important areas that contribute to health disparities: health literacy, culturally congruent care, and caregiver emotional well-being.

Our findings are consistent with other research that has found that parents of children with cancer receive inadequate attention from healthcare systems which primarily focus on the needs of the patient [9]. Spanish-speaking Latino caregivers and families are less likely to have access to psychosocial resources due to a series of systemic sociocultural barriers such as unstable employment, lack of insurance, documentation status and the unavailability of Spanish language resources [31,32]. Language itself was also found to be a significant barrier that impacts multiple areas for both the parent and patient as well as healthcare systems and providers [33]. Our community partners reported that speaking Spanish as a primary language, coupled with the medical systems’ limited Spanish resources, led to gaps in knowledge about their child’s cancer and treatment which can be observed by low levels of health literacy, difficulty understanding treatments and procedures, and seeking information from unreliable sources [14]. However, it is important to note that not all burden to increase health literacy falls on the caregiver or patient. Language barriers within healthcare systems are evident by the limited amount of information that is accessible in Spanish (i.e., discharge instructions, brochures, forms, email communication, website text), which has a detrimental impact on information transfer in populations with limited English proficiency [34]. The lack of Spanish-speaking healthcare providers despite the high percentage of Spanish-language families within the hospital system further exacerbates disparities in health literacy [35]. Limited representation within hospital systems also leads to patient–provider interactions that neglect cultural values that are important to the Latino population such as familismo, simpatía, personalismo, confianza, and respeto across multiple domains (diagnosis consults, inpatient stays, emergency room visits, outpatient services) [15].

Use of CEnR to collaboratively co-develop an intervention to address gaps in psychosocial care for Spanish-speaking Latino families impacted by childhood cancer resulted in a multimodal, culturally rich and congruent intervention intentionally designed to improve health literacy and emotional well-being in parents and caregivers that integrated cultural values and concepts to both empower families to navigate the healthcare setting and improve the experience for Latino families in the oncology setting. This collaborative work led to the successful identification of an intervention framework, the development of culturally congruent intervention components, and the ability to develop such components to be delivered using telehealth, which addresses many additional barriers to care access. Moreover, the Corazones intervention as a result of community co-development includes culturally relevant and consistent strategies of CAM, Loteria, gardening, and culinary medicine to address well-being of Spanish-speaking Latino families during children’s cancer treatment. These strategies may be overlooked in traditional intervention development approaches but have the potential to engage underrepresented communities, particularly when engaging with community experts to deliver such intervention components. The use of CEnR to co-develop a culturally relevant intervention has the potential to address cancer health disparities in underserved and minoritized populations. Engaging community partners who were bilingual and bicultural helped to address gaps in culturally relevant care with a specific focus on cultural values (e.g., familismo, simpatía, personalismo) by ensuring the resulting intervention is relevant to multiple family members, incorporates opportunities to address community needs (e.g., Q&A forums, spirituality, CAM), and creates an environment that promotes respeto with sessions conducted by community members.

The approach outlined in our work identified specific unmet community needs in the context of Spanish-speaking families impacted by childhood cancer and resulted in multiple community-relevant approaches to address these needs. This community-engaged approach ensures that the voice and perspective of the community is heard and incorporated into interventions designed to mitigate health disparities. By partnering directly with the target community, the resulting intervention not only supports cultural relevance but has implications for sustainability, particularly given that experts and providers within the target community are partners in intervention delivery. Moreover, we involved members of the cancer center of our health setting, further supporting sustainability. The goals of the intervention described in this paper include increased caregiver agency, which could potentially result in greater efficacy in navigating the healthcare system, as well as strategies to address stress, well-being, and quality of life. Healthcare experiences that are more culturally congruent can decrease caregiver stress, increase collaborative delivery of healthcare, improve treatment experience, and ideally, improve health outcomes. The intervention addresses intersectional factors contributing to health disparities, including language (intervention delivered in Spanish by native Spanish-speakers), ethnicity (intervention delivered by members of the ethnic community), socioeconomic barriers (virtual intervention mitigated needs for transportation, parking, childcare, etc.), and addressed potential for systemic racism by embedding cultural values into intervention components.

### 4.1. Limitations

Despite the contributions of this work, there are limitations of the study to be acknowledged. We recruited a convenience-based homogenous sample of low-income mothers largely from Mexico. Accordingly, generalizability of the intervention may be limited. Although we attempted to engage a broader range of parents/caregivers (e.g., fathers, grandparents) community partners who were mothers were the group who we could consistently and effectively engage. This may reflect the cultural value of marianismo, in which mothers are expected to assume primary caretaking tasks within the family [36,37]. It is also the case that systemic and structural factors place mothers in the primary role of caregiving, including factors related to gendered economic disparities, systemic biases in hiring, inadequate support for minoritized families, and lack of access to childcare and workplace flexibility/leave. Nonetheless, future efforts should ensure a diverse range of collaborative input to address generalizability across caretaker gender, socioeconomic status, and geographic origin. In addition, multiple caregivers dropped out of the CBPR engagement, largely due to the impact of having a child with cancer and dealing with the treatment experience. This may have impacted the community-engaged input into the intervention and also resulted in a smaller sample size for both community partner groups, which may have further limited generalizability. Finally, we did not collect full demographic data (e.g., marital status and education) on Phase II community partners, which may additionally limit generalizability.

### 4.2. Conclusion and Practical Implications

By partnering with Latino parents and caregivers of children with cancer, we collaboratively co-developed a behavioral intervention designed to address disparities in psychosocial functioning and quality of life for Spanish-speaking families that is culturally congruent. This process resulted in a multicomponent intervention that addresses the informational, practical, and psychosocial needs of Latino caregivers and aims to improve health literacy and emotional well-being and integrates cultural values to improve patient experience. The resulting intervention includes novel and community-specific components such as CAM strategies that focus on parent ability to provide symptom management for children, culinary medicine that includes a focus on culturally traditional recipes modified for cancer treatment considerations, and culturally relevant psychoeducational tools such as Loteria. Collaboratively and equitably engaging with community partners can be challenging, but the resulting intervention has the potential to be well-received by the community and thus may be more likely to be effective and sustainable. In addition, because the COVID-19 pandemic required us to rethink intervention delivery, the result is an intervention that addresses many barriers in access to healthcare given its virtual format (e.g., transportation, parking, child-care, geographical location), which addresses many socioeconomic barriers to healthcare access. This approach can serve as a model for addressing health equity in minoritized populations to advance cancer health disparities research. In a follow-up manuscript in this journal issue we report on formative evaluation and preliminary efficacy of the Corazones intervention.

## Figures and Tables

**Table 1:** Community partner demographics.

Demographics	Partners Phase I (n = 8)
Age (M ± SD)	36.17 ± 5.91
Sex [n (%)]	
Female	8 (100)
Education (yrs M ± SD)	12.75 ± 3.34
Partner Status [n (%)]	
Married/Partnered	5 (62.50)
Other	3 (37.50)
Country of Origin	
Mexico	5 (62.50)
Honduras	1 (12.50)
Guatemala	1 (12.50)
Not specified	1 (12.50)
Annual Household Income (US dollars, M ± SD)	20,892.67 ± 15,600.00
Number Household Members (M ± SD)	5 ± 1.73
	Partners Phase II (n = 5)
Age (M ± SD)	42.20 ± 2.87
Sex [n (%)]	
Female	5 (100)
Country of Origin	
Mexico	4 (80.00)
Guatemala	1 (20.00)
Annual Household Income (US dollars, M ± SD)	15,433.60 ± 8966.61
Number Household Members (M ± SD)	6 ± 1.50

Note. M = Mean; SD = Standard Deviation.

**Table 2:** Frequency of coded references across themes.

Themes	Coded References	N of Coded References
		Total = 59
Health Literacy	Need to understand terminology related to diagnosis and treatment.	20
	Need for information in practical terms that supports healthcare navigation.	18
	Need for information in everyday language.	21
Emotional Well-being		Total = 39
	Access to strategies to balance self- and family care in the context of cancer treatment.	16
	How to support children’s adjustment during cancer treatment.	13
	Access to resources and services (e.g., economic, social services) to support well-being.	10
Culturally Congruent Care		Total = 58
	Integration of relevant cultural values into care.	29
	Opportunities to gather health-related information in culturally congruent approaches.	12
	Strategies for self-care that are culturally congruent and in Spanish.	17

**Table 3:** Convivial cultural values in the context of U.S. healthcare practices.

Convivial/Collectivist Cultural Value	Description	Potential Conflict with U.S. Healthcare Practices/Values
Familismo	Prioritization of the family with an emphasis on emotional closeness, interdependence, and obligation that takes precedence over individual needs.	Emphasis on individual autonomy, which may conflict with family-centered decision-making.
Tacto	An expectation of gentleness, diplomacy, and emotional sensitivity in communication, particularly surrounding difficult topics.	Communication that tends to be direct and clinical, potentially perceived as blunt or insensitive.
Simpatía	Prioritization of warmth and positivity in social interactions to maintain relational harmony while avoiding conflict or negativity.	Encouragement of explicit communication of symptoms and concerns.
Confianza	A culturally congruent emphasis on mutual trust, warmth, and personal connection between families and providers.	Prioritization of efficiency and professional distance, which may weaken relationship-building preceding care.
Personalismo	A value emphasizing warm, friendly, and personal interactions that prioritize human connection over formal roles.	Interactions that are task-focused and impersonal due to time and efficiency pressures.
Respeto	A cultural norm that emphasizes respect for authority, elders, and professional roles, often involving deference.	Expectation and encouragement of patients to question providers and self-advocate, which may conflict with deference norms.
Relational decision-making/Shared responsibility	Health decisions are made collectively with family.	Prioritization of individual informed consent, privacy, and decision-making.

## Data Availability

Data presented in this paper are included in the Supplementary Materials (File S2). Further inquiries can be made to the corresponding author.

## Supplementary Materials

The following supporting information can be downloaded at: https://www.mdpi.com/article/10.3390/children13030369/s1, Figure S1. Health Literacy Brochure. Figure S2. Nutrition Label Education. Figure S3. Culturally Grounded Recipe. Figure S4. Acupressure Point and Essential Oils to Address Nausea. Figure S5. Contact Tree. Figure S6. Loteria Game Card. File S1: COREQ_Checklist. File S2: THEMES OF THE CORAZONES CANCER EXPERIENCE–PHASES I AND II.

## References

[1] Miller A., Sauer A., Ortiz A., Fedewa S., Pinheiro P., Tortolero-Luna G., Martinez-Tyson D., Jemal A., Siegel R.L. (2018). Cancer Statistics for Hispanics/Latinos, 2018. CA Cancer J. Clin..

[2] Winestone L.E., Aplenc R. (2019). Disparities in Survival and Health Outcomes in Childhood Leukemia. Curr. Hematol. Malig. Rep..

[3] Macgregor C., Walumbe J., Tulle E., Seenan C., Blane D.N. (2023). Intersectionality as a theoretical framework for researching health inequities in chronic pain. Br. J. Pain.

[4] Held M.L., Jones A., Forrest-Bank S. (2020). Predictors of Latinx Youth Health and Emotional Well-being: Social Determinants of Health Perspective. J. Racial Ethn. Health Disparities.

[5] Granek L., Rosenberg-Yunger Z.R.S., Dix D., Klaassen R.J., Sung L., Cairney J., Klassen A.F. (2014). Caregiving, single parents and cumulative stresses when caring for a child with cancer. Child Care Health Dev..

[6] Luckett T., Goldstein D., Butow P.N., Gebski V., Aldridge L.J., McGrane J., Ng W., King M.T. (2011). Psychological morbidity and quality of life of ethnic minority patients with cancer: A systematic review and meta-analysis. Lancet Oncol..

[7] Wahi A., Phelan M., Sherman-Bien S., Sender L., Fortier M. (2016). The Impact of Ethnicity, Language, and Anxiety on Quality of Life in Children with Cancer. Appl. Res. Qual. Life.

[8] Streisand R., Kazak A.E., Tercyak K.P. (2003). Pediatric-Specific Parenting Stress and Family Functioning in Parents of Children Treated for Cancer. Child. Health Care.

[9] Grant M., Sun V., Fujinami R., Sidhu R., Otis-Green S., Juarez G., Klein L., Ferrell B. (2013). Family Caregiver Burden, Skills Preparedness, and Quality of Life in Non-Small Cell Lung Cancer. Oncol. Nurs. Forum.

[10] Corà A., Partinico M., Munafò M., Palomba D. (2012). Health risk factors in caregivers of terminal cancer patients: A pilot study. Cancer Nurs..

[11] Kazak A.E., Boeving C.A., Alderfer M.A., Hwang W.T., Reilly A. (2005). Posttraumatic stress symptoms during treatment in parents of children with cancer. J. Clin. Oncol..

[12] Rodriguez E.M., Dunn M.J., Zuckerman T., Vannatta K., Gerhardt C.A., Compas B.E. (2012). Cancer-related sources of stress for children with cancer and their parents. J. Pediatr. Psychol..

[13] Sommershof A., Aichinger H., Engler H., Adenauer H., Catani C., Boneberg E.-M., Elbert T., Groettrup M., Kolassa I.-T. (2009). Substantial reduction of naïve and regulatory T cells following traumatic stress. Brain Behav. Immun..

[14] Zamora E.R., Kaul S., Kirchhoff A.C., Gwilliam V., Jimenez O.A., Morreall D.K., Montenegro R.E., Kinney A.Y., Fluchel M.N. (2016). The impact of language barriers and immigration status on the care experience for Spanish-speaking caregivers of patients with pediatric cancer. Pediatr. Blood Cancer.

[15] Campos B., Kim H.S. (2017). Incorporating the cultural diversity of family and close relationships into the study of health. Am. Psychol..

[16] Sabogal F., Marín G., Otero-Sabogal R., Marín B.V., Perez-Stable E.J. (1987). Hispanic Familism and Acculturation: What Changes and What Doesn’t?. Hisp. J. Behav. Sci..

[17] Holloway R.A., Waldrip A.M., Ickes W. (2009). Evidence that a simpático self-schema accounts for differences in the self-concepts and social behavior of Latinos versus Whites (and Blacks). J. Pers. Soc. Psychol..

[18] Acevedo A.M., Herrera C., Shenhav S., Yim I.S., Campos B. (2020). Measurement of a Latino cultural value: The Simpatía scale. Cult. Divers. Ethn. Minor. Psychol..

[19] Lopez C., Vazquez M., McCormick A.S., Gonzalez J.E., Liew J., Curtis G.A., Zou Y. (2023). Familismo, Respeto, and Bien Educado: Traditional/Cultural Models and Values in Latinos. Family Literacy Practices in Asian and Latinx Families: Educational and Cultural Considerations.

[20] Campos B., Ullman J.B., Aguilera A., Dunkel Schetter C. (2014). Familism and psychological health: The intervening role of closeness and social support. Cult. Divers. Ethn. Minor. Psychol..

[21] Meeske K.A., Sherman-Bien S., Hamilton A.S., Olson A.R., Slaughter R., Kuperberg A., Milam J. (2013). Mental health disparities between hispanic and non-hispanic parents of childhood cancer survivors. Pediatr. Blood Cancer.

[22] Mantwill S., Monestel-Umaña S., Schulz P.J. (2015). The Relationship between Health Literacy and Health Disparities: A Systematic Review. PLoS ONE.

[23] Grant S.J., Yanguela J., Odebunmi O., Grimshaw A.A., Giri S., Wheeler S.B. (2024). Systematic Review of Interventions Addressing Racial and Ethnic Disparities in Cancer Care and Health Outcomes. J. Clin. Oncol..

[24] Domenech Rodríguez M.M., Baumann A.A., Schwartz A.L. (2011). Cultural Adaptation of an Evidence Based Intervention: From Theory to Practice in a Latino/a Community Context. Am. J. Community Psychol..

[25] Israel B., Eng E., Schulz A., Parker E.A., Israel B.A., Eng E., Schulz A.J., Parker E.A. (2012). Introduction to methods in CBPR for health. Methods for Community-Based Participatory Research for Health.

[26] Wallerstein N., Duran B. (2006). Using community-based participatory research to address health disparities. Health Promot. Pract..

[27] Israel B., Schulz A., Parker E.A., Becker A.B. (1998). Review of community-based research: Assessing partnership approaches to improve public health. Annu. Rev. Public Health.

[28] Tong A., Sainsbury P., Craig J. (2007). Consolidated criteria for reporting qualitative research (COREQ): A 32-item checklist for interviews and focus groups. Int. J. Qual. Health Care..

[29] Black K.Z., Hardy C.Y., De Marco M., Ammerman A.S., Corbie-Smith G., Council B., Ellis D., Eng E., Harris B., Jackson M. (2013). Beyond incentives for involvement to compensation for consultants: Increasing equity in CBPR approaches. Prog. Community Health Partnersh. Res. Educ. Action.

[30] Weiss B.D., Mays M.Z., Martz W., Castro K.M., DeWalt D.A., Pignone M.P., Mockbee J., Hale F.A. (2005). Quick Assessment of Literacy in Primary Care: The Newest Vital Sign. Ann. Fam. Med..

[31] Zaylskie L.E., Zickafoose J.S., Leech A.A., Jennings B., Curcio N.M., Griffith K.N. (2025). Health care access, utilization, and quality for children in English versus Spanish-speaking households. Health Aff. Sch..

[32] Escobedo L.E., Cervantes L., Havranek E. (2023). Barriers in Healthcare for Latinx Patients with Limited English Proficiency—A Narrative Review. J. Gen. Intern. Med..

[33] Timmins C.L. (2002). The impact of language barriers on the health care of Latinos in the United States: A review of the literature and guidelines for practice. J. Midwifery Womens Health.

[34] Gallant L.M., Irizarry C., Boone G.M., Ruiz-Gordon B. (2010). Spanish Content on Hospital Websites: An Analysis of U.S. Hospitals’ in Concentrated Latino Communities. J. Comput.-Mediat. Commun..

[35] Fernández A., Pérez-Stable E.J. (2015). ¿Doctor, habla español? Increasing the Supply and Quality of Language-Concordant Physicians for Spanish-Speaking Patients. J. Gen. Intern. Med..

[36] Sanchez D., Vandewater E.A., Hamilton E.R. (2019). Examining marianismo gender role attitudes, ethnic identity, mental health, and substance use in Mexican American early adolescent girls. J. Ethn. Subst. Abuse..

[37] Del Gaudio F., Hichenberg S., Eisenberg M., Kerr E., Zaider T.I., Kissane D.W. (2013). Latino Values in the Context of Palliative Care: Illustrative Cases from the Family Focused Grief Therapy Trial. Am. J. Hosp. Palliat. Med..

