# Supplementary material for: A Culturally Congruent Psychosocial Intervention for Latino Caregivers of Children with Cancer: Intervention Development
Source: Children (Basel). 2026 Mar 5;13(3):369. doi: 10.3390/children13030369 (PMC13025392; doi:10.3390/children13030369)
Supplement: Supplementary file 1 [file children-13-00369-s001.zip › children-4165267-supplementary.pdf]

Supplemental Figures

Supplemental Figure S1. Health Literacy Brochure.

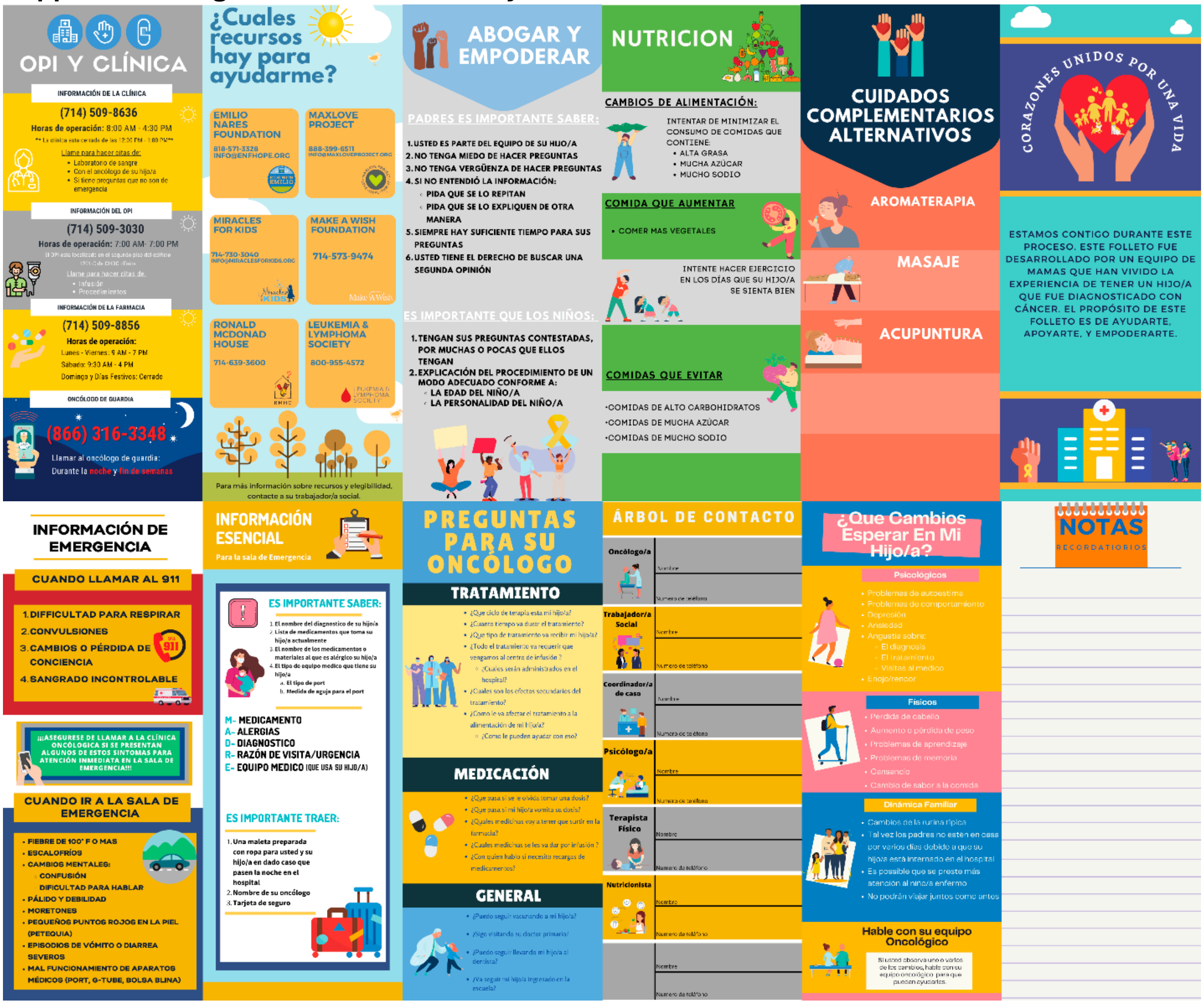

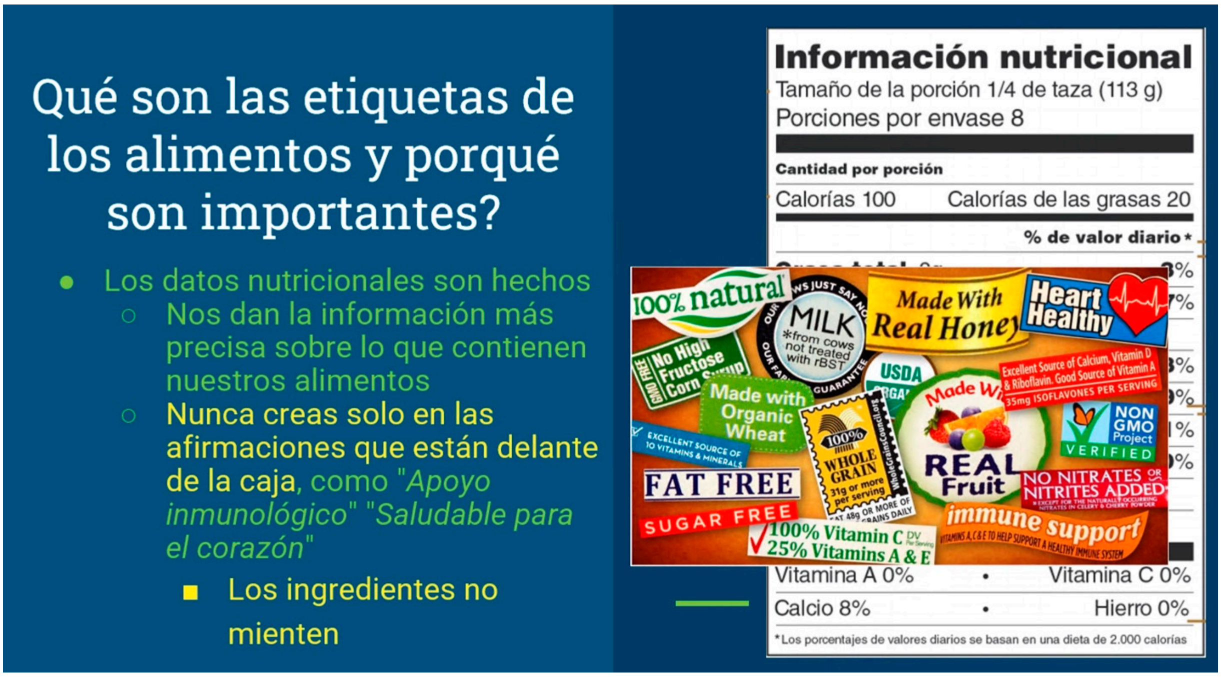

Supplemental Figure S3. Culturally Grounded Recipe.

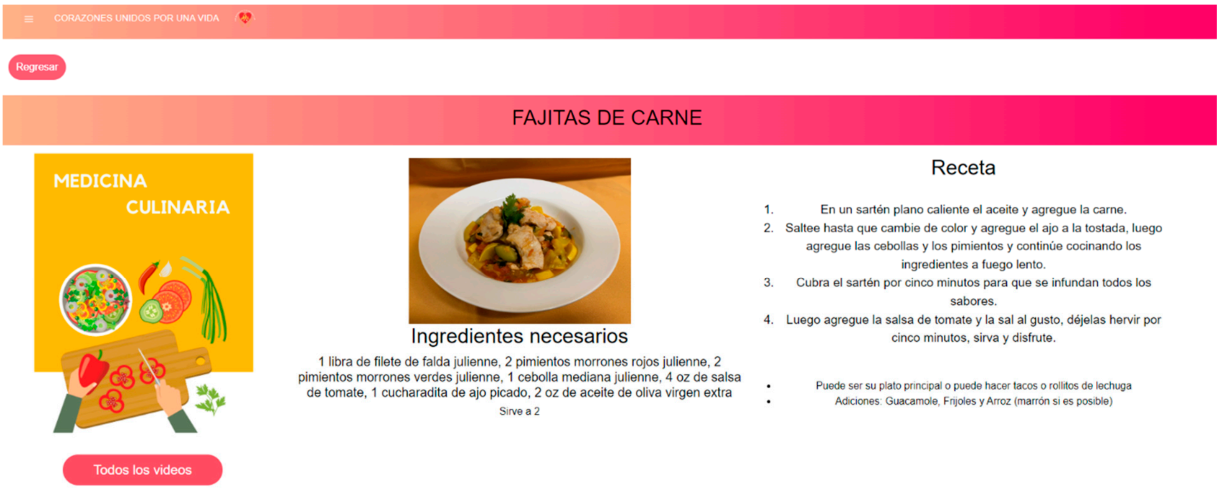

Supplemental Figure S4. Acupressure Point and Essential Oils to Address Nausea.

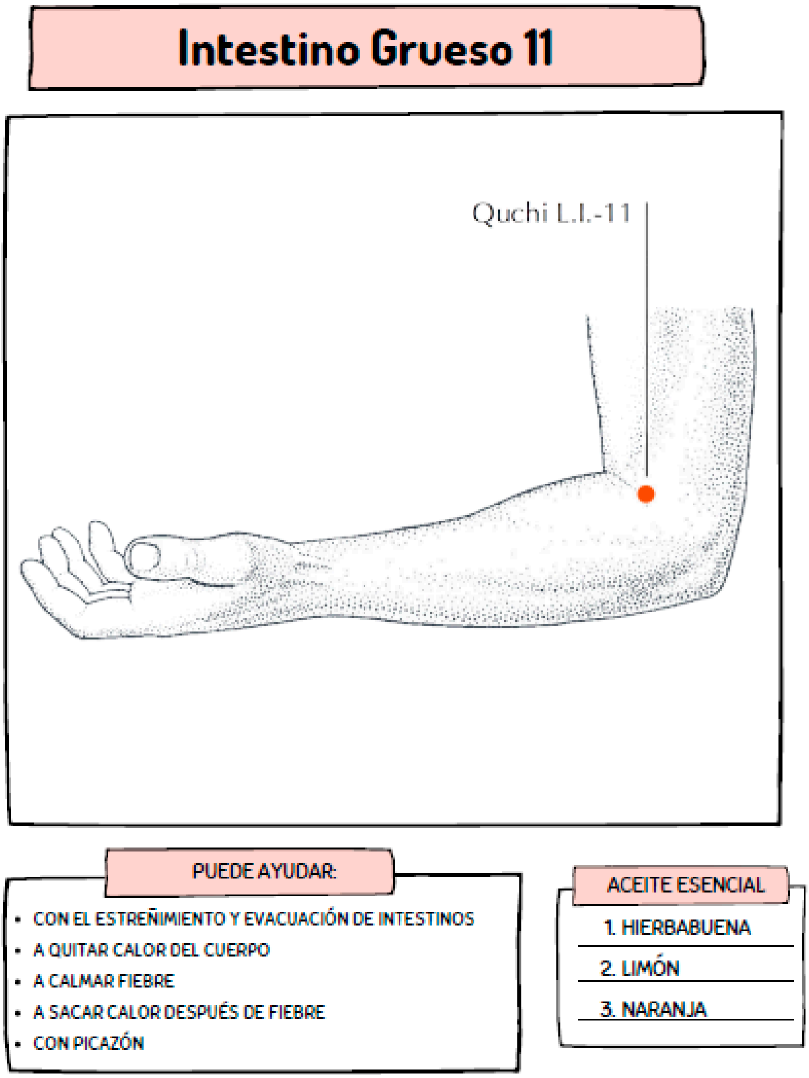

Supplemental Figure S5. Contact Tree.

ARBOL DE CONTACTO

**Oncologo/a** 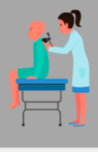	Nombre
	Numero de teléfono
**Trabajador/a Social** 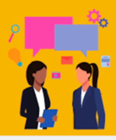	Nombre
	Numero de teléfono
**Coordinador/a de caso** 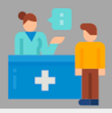	Nombre
	Numero de teléfono
**Psicólogo/a** 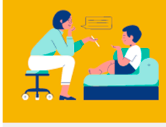	Nombre
	Numero de teléfono
**Terapista Físico** 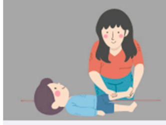	Nombre
	Numero de teléfono
**Nutricionista** 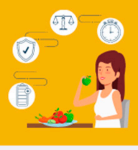	Nombre
	Numero de teléfono
**Cuidados Paliativos** 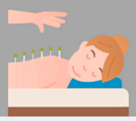	Nombre
	Numero de teléfono

Supplemental Figure S6. Loteria Game Card.

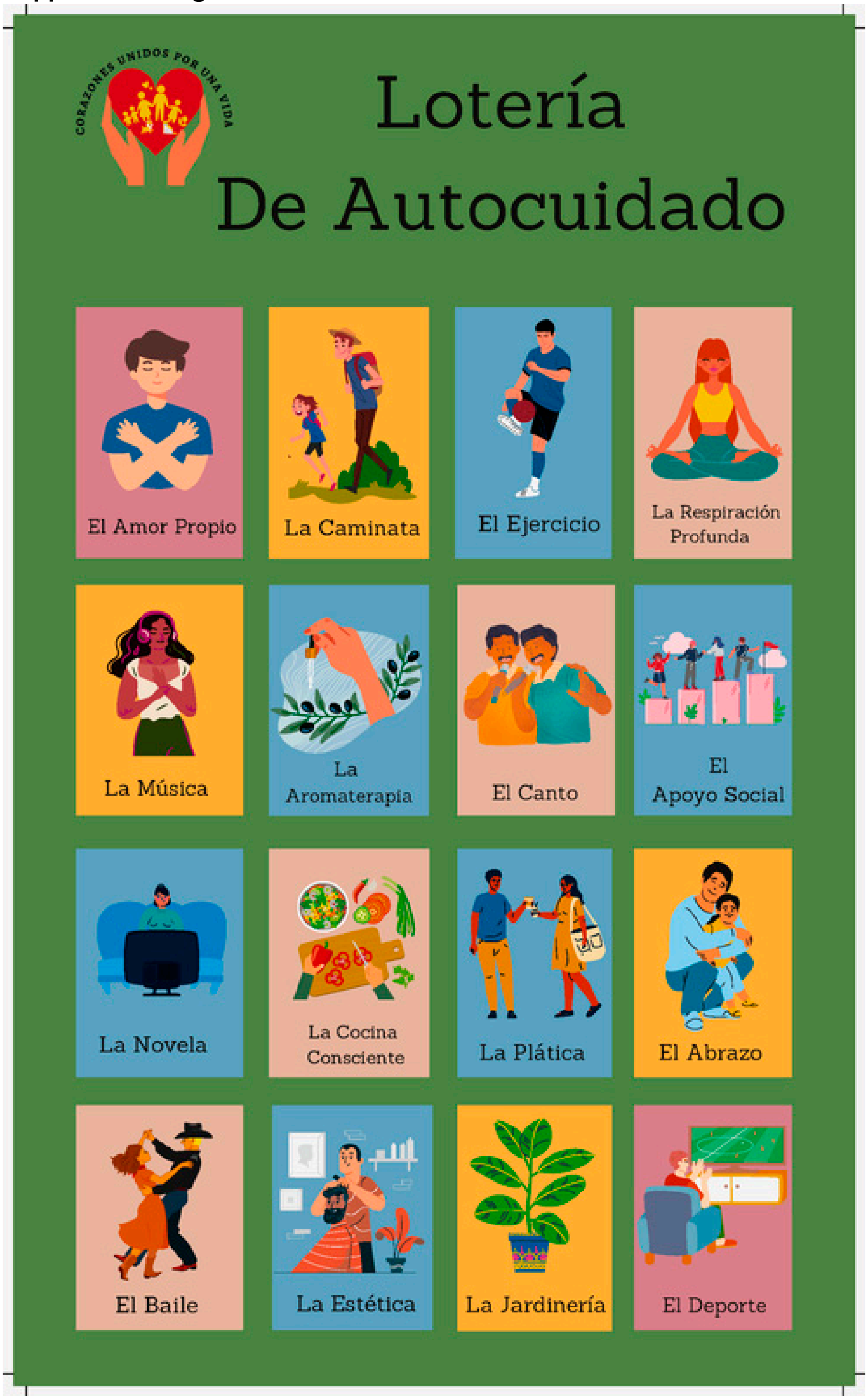

Note. The 6 supplemental figures provided are to give context for the overarching components of the intervention (i.e., health literacy, emotional well-being, and culturally congruent care). They are not meant to represent each of the individual 12 sessions of the intervention. Additional figures can be provided upon request.
