# Supplementary material for: A Culturally Congruent Psychosocial Intervention for Latino Caregivers of Children with Cancer: Intervention Development
Source: Children (Basel). 2026 Mar 5;13(3):369. doi: 10.3390/children13030369 (PMC13025392; doi:10.3390/children13030369)
Supplement: Supplementary file 1 [file children-13-00369-s001.zip › File S2.pdf]

Themes of the Corazones Cancer Experience – Phases I and II

As a collaborative group, we initially identified themes that centered on the following topics: 1) negative and traumatic experiences across various medical units (e.g., emergency department, inpatient and outpatient oncology units), 2) interactions with healthcare staff and translators that were hampered by lack of cultural competence, and 3) difficulty understanding medical processes and procedures during their child's cancer treatment. As meetings progressed and the inductive approach to coding continued, three overarching themes emerged: 1) lack of resources available focused on parent/caregiver/family health and well-being, 2) lack of health literacy, and 3) lack of culturally congruent care.

Health literacy. Health literacy is a significant factor in navigating the healthcare system, making treatment decisions, and understanding children's diagnosis, prognosis, and treatment trajectory.[1] Collaborative strategies to address health literacy among Spanish-speaking families facing a child's cancer diagnosis involved multiple strategies: distilling relevant information into a brochure families could personalize with their child's information, nutritional education in the form of culinary medicine, and question/answer sessions with oncology providers including oncologists, social workers, and case managers.

Health literacy in the context of pediatric cancer was defined by the Corazones as the understanding of medical terminology that is communicated to caregivers during interactions with healthcare providers that subsequently impacts medical decision-making ability. This operationalization is multi-dimensional and contains several underlying concepts such as adequate understanding of diagnosis, prognosis, and treatment; nutritional competencies (e.g., knowing what foods to prioritize or avoid during cancer treatment); options for and implications of

complementary and alternative interventions for supportive care during cancer treatment (e.g., herbal remedies); and navigating hospital resources and treatment options.

One member described their experience related to health literacy as: "…Doctors, at least in my case, didn't take enough time to explain, to explain the procedure well, what happens after, or what reaction they [child/patient] may have." Another member, unsure of their child's specific diagnosis, described their child's cancer (leukemia) as "the one where their blood is poisoned." Corazones often used the phrase "You don't know until you've lived it," which describes a collective experience of the community partners in which the complexities of medicine and navigating the healthcare system were not apparent until faced with a child diagnosed with cancer. The experience required quick adaptation, which was challenging without sufficient knowledge and resources to support understanding of the healthcare process and system. Complicating this process was the lack of information provided in Spanish, the lack of in-person interpreters, and the need to make decisions quickly in the context of insufficient information.

Understanding medical terminology and navigating the healthcare setting. Community partners expressed that information presented verbally, often via the use of video interpretation, was insufficient in facilitating health literacy. Moreover, the "big binder" of cancer information provided to families, often not fully in Spanish, was significantly under utilized by families. Nonetheless, all members of the Corazones indicated that written information to supplement verbal information is necessary to support health literacy. Accordingly, in order to support improved understanding of medical terminology and facilitate navigating the complex cancer healthcare environment, the Corazones and the academic researchers developed a health literacy brochure described in the manuscript to ensure families had relevant information available to them during their child's cancer treatment.

Culinary medicine. Diet and nutrition have a significant role in cancer prevention, control and survivorship.[2] Diet also varies by cultural and socioeconomic background and thus attention to specific community needs, resources, and practices was deemed essential within the Corazones. Community partners expressed that ensuring optimal nutrition across family members during a child's cancer treatment was challenging. Accordingly, the Corazones expressed a need to address nutritional information needs with a focus on understanding food labels and ingredients. This approach was chosen by the Corazones who noted that commonly used ingredients in the community previously assumed to be "healthy" often contained high amounts of sodium and sugar as well as a difficulty deciphering nutrition labels for optimal dietary intake. To complement nutrition-related health literacy, Corazones expressed a need to address cooking in a culturally responsive way to facilitate incorporating cancer-specific ways of preparing traditional recipes. Moreover, with a focus on familismo, Corazones suggested implementing cooking classes that included multiple family members to prepare the ingredients and cook a meal together that incorporated ingredients and recipes rooted in ancestral Mexican cuisine and ingredients that could be used to substitute in meals cooked at home to contain higher nutritional value. For example, one mom mentioned she learned to make a pozole (chicken or pork stew made with a variety of blended chilies and spices which contains hominy) that is lower in carbohydrates by substituting the hominy for cauliflower pieces.

Question/answer forums. Finally, our community partners shared that interactions with healthcare providers often lacked important cultural values that hampered the ability to gain necessary medical knowledge and information to guide treatment-related decision-making (see below for a full description of cultural values). For example, many community partners also noted reluctance, including embarrassment or lack of confidence, in asking healthcare providers

questions or requesting information due to perceived lack of *confianza* or *personalismo* in interactions with providers. The Corazones also noted there were few reliable and evidence-based resources, particularly Spanish-language resources, to provide information regarding their child's diagnosis, prognosis, treatment, and procedures. This substantial lack of resources was described as an additional barrier to health literacy and treatment-related decision making. A strategy to address this gap was incorporation of question/answer forums with bilingual and bicultural members of the cancer center so that families could gather relevant health information in a culturally congruent manner.

Culturally congruent care. The Corazones defined culturally grounded care as interactions with healthcare providers that incorporated important elements of Latino culture including *personalismo*, *confianza*, *respeto*, *familismo* and *simpatia*. These constructs are detailed below.

*Personalismo and confianza***.** *Personalismo* is best described as a value that places emphasis on personal interactions, which take the shape of informal and supportive structure rather than formal and professional interactions.[3] *Confianza* means trust; having relationships based on reciprocal trust where both parties have each other's best interest in mind. Interactions with elements of *personalismo* contribute to the building of *confianza.*[4]

All Corazones shared stories of the only Spanish-speaking nurse practitioner, whose bedside manner and interactions contained *personalismo* and fostered *confianza*. "…She comes and tells you everything, from my experience she is the only one who does that, she takes her time to tell you where your child is on the treatment roadmap, she knows more or less who we are as individuals. Unlike others [nurses]- they don't know who comes or goes, how long you've been coming here." The Corazones relayed that warm, interpersonal interactions were essential for

sustaining a reciprocal provider-patient relationship. The lack of bicultural providers was a significant barrier to culturally congruent and informed care.

*Respeto and familismo***.** Latino families tend to have a hierarchical culture that values *respeto*, which directly translates to respect and might be usefully conceptualized as formal politeness that is attentive to preserving the dignity of others. *Respeto* warrants different behaviors toward others based on factors such as age, gender, social or economic status, and authority.[5] *Familismo* can be described as a strong identification with the family, especially the nuclear and the extended family, as well as strong feelings of loyalty, reciprocity and solidarity among members of one same family.[6]

One member provided a hypothetical example of an interaction that contained *respeto* and *familismo* when being given their child's diagnosis as: "I think it could work something like this, when the doctor is going to share the diagnosis, perhaps they can ask 'ma'am, is someone else here with you? Do you have more family here? If you are okay with it, you can call your family members in." This highlights the importance of involving a broader range of family members in healthcare settings and discussions. In order to gain *confianza* and *respeto*, it is imperative for providers to solicit opinions from other family members who may be present to help discuss and make medical decisions.[3] Most community partners emphasized the importance of involving the family in discussions of healthcare and treatment planning and provision of treatment, which is often de-emphasized in healthcare settings.

Simpatia and tacto. Simpatia is defined as a preference for social interactions characterized by warmth and emotional positivity while also avoiding conflict and/or overt negativity.[7] The Corazones referred to this concept as "*tacto,*" or tact, which contains elements of *simpatia*. One member gave an example of a doctor who had *simpatia* and *tacto* when they were sharing the news

of her daughter's diagnosis. She said, "The doctor that I got here…was wonderful. He had a lot of tact. He brought me into a small private room in the pediatric intensive care unit (PICU), he spoke Spanish, he was very amicable. He sat in front of me and said 'ma'am, we need to share the results of your daughter's tests. Unfortunately, she has this [leukemia], we are so sorry, but we need to administer treatment immediately.' He shared the news with me in such a different manner. It didn't take too long to share the news, but he had a lot of tact in his approach."

Complementary and alternative medicine. Many community partners shared that it is common practice in Latino culture to use traditional healers such as herbalists or *sobadores* (community healers who administer massage to care for joint or muscle pain) to relieve symptoms or pain from many ailments.[8] In the academic realm, this is referred to as complementary and alternative medicine (CAM). The Corazones indicated that healthcare providers often do not ask about or focus on CAM strategies that families may be interested in or using at home. In addition, community partners reported that it is common to massage their children to relieve treatment-related pain and discomfort, however, they were often concerned about causing more pain or damage. Thus, it became clear that incorporating CAM strategies was necessary. Acupuncture and massage are common CAM strategies to integrate into a holistic approach to cancer wellness; however, our group agreed that acupuncture would not be of interest because of needle-related fears in many of the children. Collectively, our community partner collaborative agreed that acupressure, aromatherapy, and cancer-specific massage techniques would be important strategies to include in the intervention.

Understanding the culture of US medicine. In addition to CAM strategies, our community partnership described many instances of dissatisfaction and challenges in interactions in the healthcare setting that highlighted the mismatch between US healthcare culture and Latino cultural

context. Instances of interactions with providers lacking tacto and simpatia were identified as barriers to culturally congruent care that impacted family experience in the healthcare setting negatively. Thus, strategies targeting addressing the culture of US healthcare were identified as an important component to the intervention, including a brochure containing roles of oncology providers and strategies to advocate for child and family needs during cancer treatment.

Spirituality. Not originally included in the intervention conceptualized by the phase I Corazones partners, our phase II partners highlighted the importance of spirituality in their lives and in particular, when faced with a child with a life-threatening illness. In fact, all members reported religion as central in their families lives and as regularly engaging with a religious or spiritual community. Thus, it was deemed critical to incorporate a component focused on spiritual needs and functioning.

Caregiver emotional well-being. Caregiver health and well-being was defined as having a balance between the stress of caring for a child with cancer and managing daily responsibilities. Community partners shared that while their children had access to a range of healthcare resources that addressed both physical and psychosocial needs, parents and family members had access to little, if any, support. This was due to a variety of factors, including documentation status, insurance, financial status, language, and transportation barriers. One member shared her challenges achieving the balance between stress and caregiving activities: "Sometimes we are so focused on the situation and we are so stressed out that we lose focus on other things that are also important, but what takes priority is what you are living at that moment. I think that either way, we have to overcome it, learn how to manage and clearly prioritize, and leave other things behind." Adequately managing stress without available resources led the Corazones to experience guilt

when attempting to practice self-care, rather than tending to their ill child, which ultimately led to neglecting self-care.

Cultural expectations contribute to many Latinas internalizing the value of *marianismo*, which refers to the gender role expectations influenced by qualities ascribed to the Virgin Mary. As such, expectations include being self-sacrificing, dedicated, and supporting wives and mothers, and being ready to help those in need in both the family and the community.[3,4] Consistent with this value, the Corazones described not having the time or ability to process medical and treatment decisions in the context of the emotional burden of having a child with a life-threatening illness. In addition to not having sufficient time or resources to cope, they expressed that their health and well-being was also being significantly impacted by employment loss, unstable financial security, and unreliable transportation to and from appointments because of assuming the head caregiver role. One community partner described challenges she faced caring for her ill child while her partner fulfilled head-of-household financial duties: "My husband does not come [to appointments] anymore. I am the one who always comes because if my husband comes, things get more stressful." All Corazones shared that their consistent choice to prioritize the needs of their sick and healthy children, household duties, and employment (or lack of) leads to burnout. Because of the challenges in engaging in self-care, the conclusion was there was a need for strategies to reduce emotional distress that focused on the family, rather than the individual parent/caregiver.

Psychoeducation. Many community members expressed a need for more intensive psychosocial follow-up throughout their children's treatment journey. Multiple Corazones observed behavioral or emotional impacts on their children because of their cancer experience. The Corazones shared that their consistent choice to prioritize the needs of the sick and healthy children, household duties and employment leads to burnout, and guilt when taking time to address

their mental, physical or emotional needs. One member stated, “When we are told the word cancer, we automatically associate it with death and we need to work on reframing that.” Community members recognized the importance of preparing and supporting children emotionally and behaviorally through treatment to prevent future negative sequelae.

Gardening. When conceptualizing a stress relieving activity that the family can do together, consistent with the cultural value of familismo, virtual gardening lessons were proposed. Because cancer patients are immunocompromised, spending time outdoors is often restricted. Gardening is an opportunity to be outside while maintaining social distancing practices. Moreover, evidence supports that the practice of gardening reduces stress.[9] Many community members stated that they currently grow many of their vegetables and their children enjoy helping in that process, further supporting gardening as an intervention component to target emotional well-being.

Dance-based movement. The Corazones acknowledged a need for a component focused on self-care that included physical fitness/movement but stressed that traditional interventions in the context of cancer caregiving (i.e., yoga[10–13]) were not preferred, which is consistent with evidence showing that yoga is a preferred practice among largely White and Asian-American populations and individuals with high socioeconomic status (SES) including years of education and income.[14] Instead, culturally congruent exercise, such as dance-based fitness, was preferred. In particular, dance-based movement can be done as a family, can incorporate culturally congruent music, and was described as a social activity that could reduce stress and improve emotional well-being.

Summary. The partnership with the Corazones highlighted the significant mismatch between Latino collectivist culture that prioritizes family relationships over the self; warm and positive social interactions; respect and tact in communication; and trust in relationships, all which can be lacking in interactions in Western medicine. The Corazones expressed that they desired

interactions with healthcare providers in which tone took precedent over spoken content; interactions with *simpatia* make parents and children feel *respetados* (respected). Feeling *respetado* may also lead to a greater sense of *confianza* in the healthcare providers, which may allow parents to feel empowered to take agency in their child's healthcare and create and strengthen rapport with the clinical team, thus supporting treatment-related decision-making The lack of culturally congruent care accessible to Spanish-speaking populations can lead to caregivers having less confidence in their child's treatment recommendations and being less likely to ask follow-up questions or voice concerns.[15] This sustained partnership highlighted specific unmet psychosocial needs of Spanish-speaking Latino parents and caregivers, including health literacy, strategies to improve emotional well-being, and the need to culturally informed care. As a result, through this partnership we conceptualized and developed a 12 session behavioral intervention which we subsequently evaluated for feasibility, relevance, likeability, and preliminary efficacy.

.

1. Al Hussein Al Awamlh B, Moses KA, Whitman J, Stewart T, Kripalani S, Idrees K. Health literacy and all-cause mortality among cancer patients. *Cancer*. 2025;131(6):e35794. doi:10.1002/cncr.35794

2. Doyle C, Kushi LH, Byers T, et al. Nutrition and Physical Activity During and After Cancer Treatment: An American Cancer Society Guide for Informed Choices. *CA Cancer J Clin*. 2006;56(6):323-353. doi:10.3322/canjclin.56.6.323

3. Carteret M. Cultural Values of Latino Patients and Families. http://www.dimensionsofculture.com/ 2011/03/cultural-values-of-latino-patients-and-families/. 2011.

4. Hausmann-Stabile C, Zayas LH, Runes S, Abenis-Cintron A, Calzada E. Ganando Confianza: Research Focus Groups with Immigrant Mexican Mothers. *Educ Train Autism Dev Disabil*. 2011;46(1):3-10. doi:10.1038/jid.2014.371

5. Lopez C, Vazquez M, McCormick AS. Familismo, Respeto, and Bien Educado: Traditional/Cultural Models and Values in Latinos. In: Gonzalez JE, Liew J, Curtis GA, Zou Y, eds. *Family Literacy Practices in Asian and Latinx Families: Educational and Cultural Considerations*. Springer International Publishing; 2023:87-102. doi:10.1007/978-3-031-14470-7_6

6. Triandis H, Marin G, Betancourt H, Lisansky J, Chang B. *Dimensions of Familism among Hispanic and Mainstream Navy Recruits*. Department of Psychology, University of Illinois; 1982:1-16.

7. Acevedo, A. M., Herrera C, Shenhav S, Yim IS, Campos B. Measurement of a Latino cultural value: The Simpatía scale. *Cultur Divers Ethnic Minor Psychol*. 2020;26(4):419-425.

8. Sandberg JC, Quandt SA, Graham A, Stub T, Mora DC, Arcury TA. Medical Pluralism in the Use of Sobadores among Mexican Immigrants to North Carolina. *J Immigr Minor Health*. 2018;20(5):1197-1205. doi:10.1016/j.physbeh.2017.03.040

9. Van Den Berg AE, Custers MHG. Gardening promotes neuroendocrine and affective restoration from stress. *J Health Psychol*. 2011;16(1):3-11. doi:10.1177/1359105310365577

10. Martin AC, Keats MR. The impact of yoga on quality of life and psychological distress in caregivers for patients with cancer. *Oncol Nurs Forum*. 2014;41(3):257-264. doi:10.1188/14.ONF.257-264

11. Sloman R. Relaxation and imagery for anxiety and depression control in community patients with advanced cancer. *Cancer Nurs*. 2002;25(6):432-435. doi:10.1097/00002820-200212000-00005

12. Baider L, Uziely B, Kaplan De-Nour A. Progressive Muscle Relaxation and Guided Imagery in cancer patients. *Gen Hosp Psychiatry*. 1994;16(5):340-347. doi:10.1016/0163-8343(94)90021-3

13. Rao R, Amritanshu R, Vinutha H, et al. Role of Yoga in Cancer Patients: Expectations, Benefits, and Risks: A Review Raghavendra. *Indian J Palliat Care*. 2017;23(3):225-230. doi:10.4103/IJPC.IJPC_107_17

14. Park CL, Braun T, Siegel T. Who practices yoga? A systematic review of demographic, health-related, and psychosocial factors associated with yoga practice. *J Behav Med*. 2015;38(3):460-471. doi:10.1007/s10865-015-9618-5

15. Zamora ER, Kaul S, Kirchhoff AC, et al. The impact of language barriers and immigration status on the care experience for Spanish-speaking caregivers of patients with pediatric cancer. *Pediatr Blood Cancer*. Published online 2016. doi:10.1002/pbc.26150
